# Crystal structure of tris­(μ-bis­{4-[(pyridin-2-yl­methyl­idene)amino]­phen­yl}methane-κ^4^ *N*,*N*′:*N*′′,*N*′′′)dizinc tetra­kis­(tetra­fluorido­borate) aceto­nitrile tris­olvate

**DOI:** 10.1107/S205698901502455X

**Published:** 2015-12-31

**Authors:** Maria-Gabriela Alexandru, Florina Dumitru

**Affiliations:** a Faculty of Applied Chemistry and Materials Science, University Politehnica of Bucharest, Polizu 1, 011061 Bucharest, Romania

**Keywords:** crystal structure, Zn^II^ complex, triple-helical motif

## Abstract

The asymmetric unit of the title compound, [Zn_2_(C_25_H_20_N_4_)_3_](BF_4_)_4_·3CH_3_CN, consists of one dinuclear Zn^II^ complex cation with a triple-helical [Zn_2_ *L*_3_]^4+^ motif (*L* is bis­{4-[(pyridin-2-yl­methyl­idene)amino]­phen­yl}methane), four BF_4_^−^ anions and three CH_3_CN solvent mol­ecules. The Zn⋯Zn separation is 11.3893 (14) Å and the ligands wrap around the two Zn^II^ atoms, forming a triple helix as defined by the Zn—N—N—Zn torsion angles of 104.05 (18), 99.06 (19) and 101.40 (19)°. The Zn—N(pyrid­yl) distances in the octahedral ZnN_6_ coordination sphere are in the range 2.128 (5)–2.190 (5) Å and the Zn—N(imine) distances are in the range 2.157 (5)–2.277 (5) Å.

## Related literature   

Other dinuclear triple-helical complexes of divalent transition metal ions with this ditopic ligand are: [Ni_2_(C_25_H_20_N_4_)_3_](BF_4_)_4_·2CH_3_OH (Hannon *et al.*, 1997 ▸), [Zn_2_(C_25_H_20_N_4_)_3_](ClO_4_)_4_·DMF·2CH_3_CN (Noboru & Kazuhiko, 1997 ▸), [Co_2_(C_25_H_20_N_4_)_3_](NO_3_)_4_·8H_2_O (Xu *et al.*, 2001 ▸), [Cu_2_(C_25_H_20_N_4_)_3_](ClO_4_)_4_·3CH_3_CN (Keegan *et al.*, 2002 ▸), [Ru_2_(C_25_H_20_N_4_)_3_](PF_6_)_4_·0.5CH_3_OH·0.5H_2_O·C_6_H_6_ (Pascu *et al.*, 2007 ▸) and [Fe_2_(C_25_H_20_N_4_)_3_]*X*_4_ [*X* = Cl^−^ (Kerckhoffs *et al.*, 2007 ▸), ClO_4_^−^ (Young *et al.*, 2013 ▸) and BF_4_^−^ as the 3.5H_2_O adduct (Vellas *et al.*, 2013 ▸)]. For the synthesis of the ligand, see: Noboru & Kazuhiko (1997 ▸); Dehghanpour *et al.* (2010 ▸).
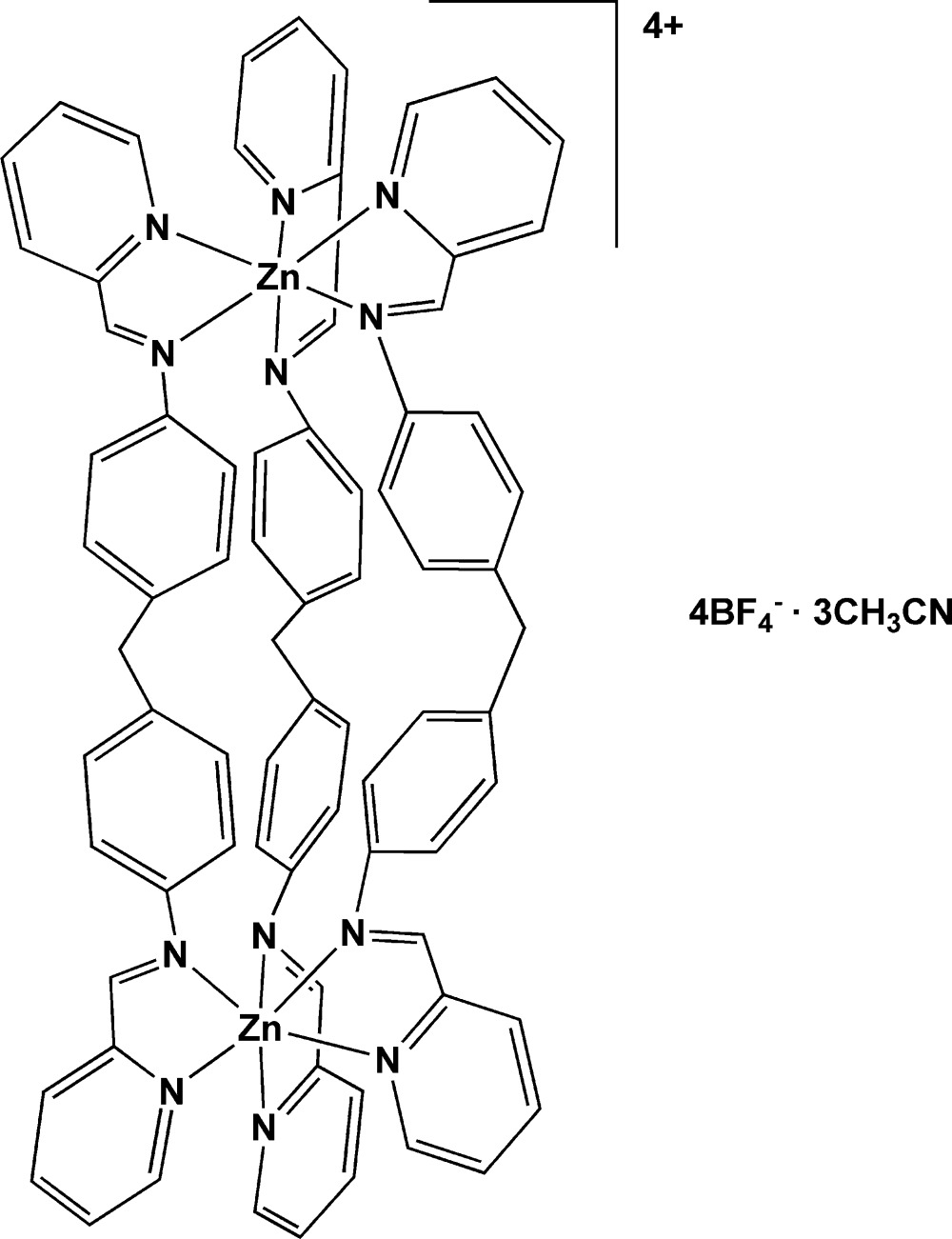


## Experimental   

### Crystal data  

[Zn_2_(C_25_H_20_N_4_)_3_](BF_4_)_4_·3C_2_H_3_N
*M* *_r_* = 1730.49
Monoclinic, 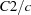

*a* = 54.177 (4) Å
*b* = 13.6929 (9) Å
*c* = 21.9343 (14) Å
β = 95.713 (1)°
*V* = 16191.0 (18) Å^3^
*Z* = 8
Mo *K*α radiation
μ = 0.68 mm^−1^
*T* = 297 K
0.41 × 0.30 × 0.26 mm

### Data collection  

Bruker SMART CCD area-detector diffractometer
Absorption correction: multi-scan (*SADABS*; Sheldrick, 2004 ▸) *T*_min_ = 0.767, *T*_max_ = 0.842
85662 measured reflections
16566 independent reflections
11969 reflections with *I* > 2σ(*I*)
*R*_int_ = 0.108

### Refinement  

*R*[*F* ^2^ > 2σ(*F* ^2^)] = 0.116
*wR*(*F* ^2^) = 0.214
*S* = 1.21
16566 reflections
1094 parameters
H-atom parameters constrained
Δρ_max_ = 0.53 e Å^−3^
Δρ_min_ = −0.52 e Å^−3^

### 

Data collection: *SMART* (Bruker, 2012 ▸); cell refinement: *SAINT* (Bruker, 2012 ▸); data reduction: *SAINT*; program(s) used to solve structure: *SHELXT* (Sheldrick, 2015*a* ▸); program(s) used to refine structure: *SHELXL2014* (Sheldrick, 2015*b* ▸); molecular graphics: *DIAMOND* (Brandenburg, 1999 ▸); software used to prepare material for publication: *publCIF* (Westrip, 2010 ▸).

## Supplementary Material

Crystal structure: contains datablock(s) I, New_Global_Publ_Block. DOI: 10.1107/S205698901502455X/fk2093sup1.cif

Structure factors: contains datablock(s) I. DOI: 10.1107/S205698901502455X/fk2093Isup2.hkl

**Fig. 1.**
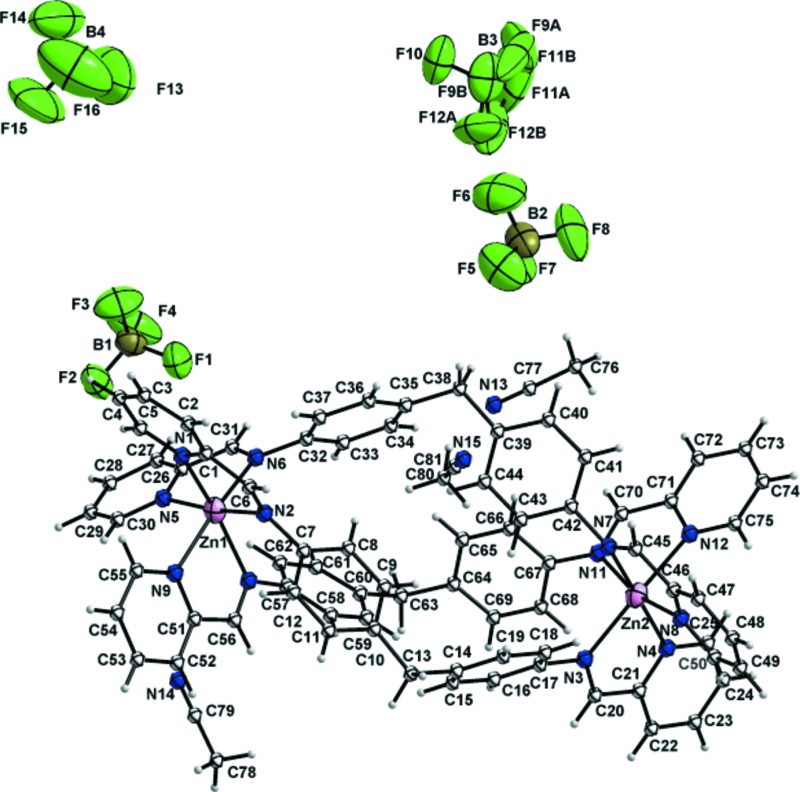
Molecular structure of the title compound. Anisotropic displacement ellipsoids are drawn at the 50% probability level and H atoms are represented by circles of arbitrary size.

. DOI: 10.1107/S205698901502455X/fk2093fig1.tif
Mol­ecular structure of the title compound. Anisotropic displacement ellipsoids are drawn at the 50% probability level and H atoms are represented by circles of arbitrary size.

CCDC reference: 1443648

Additional supporting information:  crystallographic information; 3D view; checkCIF report

## supplementary crystallographic information

### S1. Introduction

In the title compound, the bis­(pyridyl­imine) ligand containing the di­phenyl­methane spacer {*L* = C_25_H_20_N_4_, systematic name: (7E)-4-((*E*)-4-((pyridin-2-yl)methyl­ene­amino)- benzyl)-*N*-((pyridin-2-yl)methyl­ene)benzenamine} adopts an angular conformation yielding a dinuclear triple helicate structure.

### S2.1. Synthesis and crystallization

Schiff base, bis­(4-(2-pyridyl­methyl­ene­amino­phenyl)­methane (*L*= C_25_H_20_N_4_), was prepared from 4,4'-di­amino­diphenyl­methane (396 mg, 2 mmol) and 2-pyridine­carboxaldehyde (380 µ*L*, 4 mmol) in aceto­nitrile, refluxed under constant stirring for 2 h. Synthetic procedures for this ligand are already described in the literature. Noboru & Kazuhiko (1997), Dehghanpour *et al.* (2010).

^1^H-NMR (δ, DMSO-d_6_, 300 MHz): 8.72–8.70 (2*H*, d, H^pyridyl^), 8.59 (2*H*, s, –CH=N–), 8.16–8.13 (2*H*, d, H^pyridyl^), 7.97–7.91 (2*H*, dt, H^pyridyl^), 7.54–7.50 (2*H*, dt, H^pyridyl^), 7.32–7.31 (8*H*, s, s, amino­phenyl), 4.01 (2*H*, s, –CH_2_–).

376 mg (1 mmol) bis­(4-(2-pyridyl­methyl­ene­amino­phenyl)­methane and 160 mg (0.67 mmol) Zn(BF_4_)_2_·xH_2_O were stirred in 5 ml CH_3_CN at 60°C for 1 h. Layering the solution of complex in CH_3_CN with iso­propyl­ether at room temperature afforded yellow crystals suitable for X-ray single-crystal experiments.

### S2.2. Refinement

The H atoms were derived from geometrical considerations and refined at idealized positions using a riding model, with C—H = 0.93–0.97 Å, *U*_iso_(H)= 1.2*U*_eq_(C) and *U*_iso_(H)= 1.5*U*_eq_(C_methyl_). Methyl-H atom positions from Fourier maps (HFIX 137) and refined as before. BF_4_ anions are disordered to some degree. For the most pronounced (B3 group), a split model has been applied with site occupation factors 0.5 for F9, F11 and F12 each. Due to various disorder problems of BF_4_ and aceto­nitrile moieties and thus mean crystal quality, data suffer from these problems but give acceptable refinement results.

### S3. Results and discussion

The asymmetric unit of the title complex is composed from one dinuclear homometallic Zn^II^ complex cation with a triple-helical motif, [Zn_2_*L*_3_]^4+^, four BF_4_^−^ anions and three aceto­nitrile solvent molecules. Each Zn^II^ metal ion is coordinated by three imino and three pyridine nitro­gen atoms, from three Schiff base ligands. The coordination geometry of the Zn^II^ metal ions is best described as slightly distorted o­cta­hedral. Zn–N(pyridyl) distances are in the range 2.128 (5) and 2.190 (5) Å and Zn–*N*(imine) distances in the range 2.157 (5) and 2.277 (5) Å. For Zn1, the torsion angle of the equatorial plane, N1–N6–N10–N9 is of 9.45 (18)° and the angle involving the apical N atoms, N2–Zn1–N5 is of 172.79 (18)°. For Zn2, the value of the torsion angle N12–N11–N3–N8, in the equatorial plane, is 10.65 (19)° and the angle formed by the apical N atoms and Zn2, N4–Zn2–N7 is 174.04 (17)°. The Zn1—Zn2 distance is 11.3893 (14) Å and, in terms of their supra­molecular cation [Zn_2_*L*_3_]^4+^, (*L* = C_25_H_20_N_4_),the structure is very similar to [Zn_2_(C_25_H_20_N_4_)_3_][ClO_4_]_4_·DMF·2CH_3_CN, Noboru & Kazuhiko (1997).

### Crystal data

**Table:**

[Zn_2_(C_25_H_20_N_4_)_3_](BF_4_)_4_·3C_2_H_3_N	*F*(000) = 7072
*M**_r_* = 1730.49	*D*_x_ = 1.420 Mg m^−^^3^
Monoclinic, *C*2/*c*	Mo *K*α radiation, λ = 0.71073 Å
*a* = 54.177 (4) Å	Cell parameters from 4655 reflections
*b* = 13.6929 (9) Å	θ = 2.3–17.9°
*c* = 21.9343 (14) Å	µ = 0.68 mm^−^^1^
β = 95.713 (1)°	*T* = 297 K
*V* = 16191.0 (18) Å^3^	Block, yellow
*Z* = 8	0.41 × 0.30 × 0.26 mm

### Data collection

**Table:**

Bruker SMART CCD area-detector diffractometer	16566 independent reflections
Radiation source: fine-focus sealed tube	11969 reflections with *I* > 2σ(*I*)
Graphite monochromator	*R*_int_ = 0.108
phi and ω scans	θ_max_ = 26.4°, θ_min_ = 0.8°
Absorption correction: multi-scan (*SADABS*; Sheldrick, 2004)	*h* = −67→67
*T*_min_ = 0.767, *T*_max_ = 0.842	*k* = −17→17
85662 measured reflections	*l* = −27→27

### Refinement

**Table:**

Refinement on *F*^2^	Primary atom site location: structure-invariant direct methods
Least-squares matrix: full	Secondary atom site location: difference Fourier map
*R*[*F*^2^ > 2σ(*F*^2^)] = 0.116	Hydrogen site location: difference Fourier map
*wR*(*F*^2^) = 0.214	H-atom parameters constrained
*S* = 1.21	*w* = 1/[σ^2^(*F*_o_^2^) + (0.0492*P*)^2^ + 80.9103*P*] where *P* = (*F*_o_^2^ + 2*F*_c_^2^)/3
16566 reflections	(Δ/σ)_max_ = 0.006
1094 parameters	Δρ_max_ = 0.53 e Å^−^^3^
0 restraints	Δρ_min_ = −0.52 e Å^−^^3^

### Special details

**Table:**

Geometry. All e.s.d.'s (except the e.s.d. in the dihedral angle between two l.s. planes) are estimated using the full covariance matrix. The cell e.s.d.'s are taken into account individually in the estimation of e.s.d.'s in distances, angles and torsion angles; correlations between e.s.d.'s in cell parameters are only used when they are defined by crystal symmetry. An approximate (isotropic) treatment of cell e.s.d.'s is used for estimating e.s.d.'s involving l.s. planes.

### Fractional atomic coordinates and isotropic or equivalent isotropic displacement parameters (Å^2^)

**Table:**

	*x*	*y*	*z*	*U*_iso_*/*U*_eq_	Occ. (<1)
Zn1	0.18372 (2)	0.80748 (5)	0.26782 (3)	0.03879 (19)	
Zn2	0.06562 (2)	0.21303 (5)	0.02048 (3)	0.03821 (19)	
C1	0.15065 (11)	0.9825 (4)	0.2709 (3)	0.0385 (14)	
C2	0.14076 (13)	1.0703 (5)	0.2855 (3)	0.0510 (17)	
H2	0.1271	1.0952	0.2614	0.061*	
C3	0.15096 (15)	1.1222 (5)	0.3359 (3)	0.0595 (19)	
H3	0.1446	1.1826	0.3458	0.071*	
C4	0.17071 (14)	1.0816 (5)	0.3710 (3)	0.061 (2)	
H4	0.1781	1.1142	0.4053	0.074*	
C5	0.17964 (12)	0.9903 (5)	0.3544 (3)	0.0493 (16)	
H5	0.1928	0.9625	0.3790	0.059*	
C6	0.14206 (11)	0.9287 (4)	0.2156 (3)	0.0423 (15)	
H6	0.1295	0.9544	0.1884	0.051*	
C7	0.14522 (10)	0.8007 (4)	0.1460 (3)	0.0389 (14)	
C8	0.13467 (12)	0.7090 (4)	0.1431 (3)	0.0495 (16)	
H8	0.1306	0.6781	0.1785	0.059*	
C9	0.13021 (12)	0.6632 (4)	0.0866 (3)	0.0475 (16)	
H9	0.1223	0.6029	0.0844	0.057*	
C10	0.13715 (11)	0.7046 (4)	0.0340 (3)	0.0388 (14)	
C11	0.14792 (13)	0.7961 (5)	0.0388 (3)	0.0530 (17)	
H11	0.1527	0.8260	0.0038	0.064*	
C12	0.15180 (13)	0.8442 (4)	0.0939 (3)	0.0509 (17)	
H12	0.1589	0.9062	0.0958	0.061*	
C13	0.13408 (13)	0.6540 (5)	−0.0280 (3)	0.0508 (17)	
H13A	0.1224	0.6912	−0.0552	0.076*	
H13B	0.1499	0.6549	−0.0451	0.076*	
C14	0.12513 (11)	0.5495 (4)	−0.0266 (2)	0.0369 (14)	
C15	0.14103 (11)	0.4751 (5)	−0.0070 (3)	0.0443 (15)	
H15	0.1576	0.4894	0.0049	0.053*	
C16	0.13293 (11)	0.3792 (4)	−0.0044 (3)	0.0435 (15)	
H16	0.1439	0.3297	0.0090	0.052*	
C17	0.10857 (10)	0.3587 (4)	−0.0221 (2)	0.0338 (13)	
C18	0.09219 (10)	0.4322 (4)	−0.0421 (3)	0.0361 (13)	
H18	0.0756	0.4178	−0.0537	0.043*	
C19	0.10065 (12)	0.5277 (4)	−0.0447 (3)	0.0417 (15)	
H19	0.0897	0.5771	−0.0587	0.050*	
C20	0.11083 (11)	0.1914 (4)	−0.0413 (3)	0.0376 (14)	
H20	0.1255	0.2044	−0.0586	0.045*	
C21	0.10161 (11)	0.0913 (4)	−0.0384 (3)	0.0368 (14)	
C22	0.11418 (13)	0.0141 (5)	−0.0610 (3)	0.0538 (17)	
H22	0.1292	0.0235	−0.0775	0.065*	
C23	0.10385 (14)	−0.0778 (5)	−0.0584 (4)	0.066 (2)	
H23	0.1118	−0.1314	−0.0737	0.079*	
C24	0.08229 (15)	−0.0898 (5)	−0.0337 (4)	0.066 (2)	
H24	0.0752	−0.1514	−0.0318	0.079*	
C25	0.07087 (12)	−0.0088 (5)	−0.0111 (3)	0.0539 (18)	
H25	0.0561	−0.0176	0.0066	0.065*	
C26	0.20789 (12)	0.7142 (5)	0.3785 (3)	0.0464 (16)	
C27	0.22505 (14)	0.6791 (5)	0.4233 (3)	0.060 (2)	
H27	0.2208	0.6316	0.4507	0.071*	
C28	0.24878 (15)	0.7159 (6)	0.4268 (4)	0.067 (2)	
H28	0.2609	0.6930	0.4563	0.080*	
C29	0.25425 (13)	0.7855 (7)	0.3867 (4)	0.071 (2)	
H29	0.2701	0.8123	0.3891	0.086*	
C30	0.23621 (12)	0.8169 (6)	0.3422 (3)	0.0613 (19)	
H30	0.2402	0.8644	0.3146	0.074*	
C31	0.18258 (12)	0.6756 (4)	0.3708 (3)	0.0438 (15)	
H31	0.1777	0.6293	0.3981	0.053*	
C32	0.14314 (11)	0.6615 (4)	0.3180 (2)	0.0368 (13)	
C33	0.13971 (12)	0.5610 (4)	0.3190 (3)	0.0430 (15)	
H33	0.1532	0.5195	0.3269	0.052*	
C34	0.11648 (13)	0.5239 (5)	0.3082 (3)	0.0509 (17)	
H34	0.1144	0.4566	0.3100	0.061*	
C35	0.09558 (11)	0.5825 (5)	0.2944 (3)	0.0422 (15)	
C36	0.09931 (12)	0.6819 (5)	0.2948 (3)	0.0458 (15)	
H36	0.0857	0.7232	0.2873	0.055*	
C37	0.12263 (11)	0.7218 (4)	0.3060 (2)	0.0393 (14)	
H37	0.1246	0.7892	0.3054	0.047*	
C38	0.07006 (12)	0.5391 (5)	0.2802 (3)	0.0538 (18)	
H38A	0.0579	0.5899	0.2845	0.081*	
H38B	0.0677	0.4892	0.3105	0.081*	
C39	0.06486 (11)	0.4940 (4)	0.2169 (3)	0.0399 (14)	
C40	0.04242 (13)	0.4487 (5)	0.2004 (3)	0.0597 (19)	
H40	0.0305	0.4487	0.2281	0.072*	
C41	0.03687 (12)	0.4034 (5)	0.1443 (3)	0.0563 (18)	
H41	0.0216	0.3735	0.1347	0.068*	
C42	0.05422 (11)	0.4033 (4)	0.1032 (3)	0.0390 (14)	
C43	0.07670 (12)	0.4483 (5)	0.1177 (3)	0.0491 (16)	
H43	0.0885	0.4482	0.0896	0.059*	
C44	0.08185 (12)	0.4938 (4)	0.1739 (3)	0.0468 (16)	
H44	0.0971	0.5247	0.1829	0.056*	
C45	0.03174 (12)	0.3783 (4)	0.0085 (3)	0.0441 (15)	
H45	0.0214	0.4284	0.0190	0.053*	
C46	0.02695 (11)	0.3291 (4)	−0.0513 (3)	0.0412 (14)	
C47	0.00989 (13)	0.3640 (5)	−0.0967 (3)	0.0585 (19)	
H47	0.0006	0.4194	−0.0901	0.070*	
C48	0.00685 (15)	0.3158 (6)	−0.1520 (3)	0.072 (2)	
H48	−0.0045	0.3380	−0.1835	0.087*	
C49	0.02088 (15)	0.2342 (6)	−0.1596 (3)	0.070 (2)	
H49	0.0190	0.2000	−0.1964	0.084*	
C50	0.03765 (13)	0.2033 (6)	−0.1127 (3)	0.0600 (19)	
H50	0.0472	0.1483	−0.1186	0.072*	
C51	0.21220 (12)	0.8324 (5)	0.1592 (3)	0.0522 (17)	
C52	0.22358 (16)	0.8724 (6)	0.1120 (4)	0.080 (3)	
H52	0.2276	0.8339	0.0795	0.095*	
C53	0.22903 (17)	0.9718 (6)	0.1138 (4)	0.089 (3)	
H53	0.2369	1.0011	0.0826	0.107*	
C54	0.22260 (16)	1.0253 (5)	0.1622 (4)	0.079 (3)	
H54	0.2262	1.0917	0.1647	0.095*	
C55	0.21086 (13)	0.9806 (5)	0.2073 (4)	0.0601 (19)	
H55	0.2065	1.0181	0.2400	0.072*	
C56	0.20671 (12)	0.7279 (5)	0.1614 (3)	0.0514 (17)	
H56	0.2103	0.6884	0.1289	0.062*	
C57	0.19399 (11)	0.5857 (4)	0.2052 (3)	0.0427 (15)	
C58	0.18393 (13)	0.5363 (5)	0.1534 (3)	0.0567 (18)	
H58	0.1796	0.5703	0.1172	0.068*	
C59	0.18044 (14)	0.4370 (5)	0.1556 (3)	0.062 (2)	
H59	0.1733	0.4047	0.1209	0.075*	
C60	0.18731 (11)	0.3841 (4)	0.2080 (3)	0.0456 (16)	
C61	0.19820 (12)	0.4328 (4)	0.2580 (3)	0.0471 (16)	
H61	0.2035	0.3980	0.2933	0.057*	
C62	0.20145 (12)	0.5326 (5)	0.2568 (3)	0.0501 (16)	
H62	0.2088	0.5644	0.2915	0.060*	
C63	0.18260 (12)	0.2747 (4)	0.2095 (4)	0.0567 (18)	
H63A	0.1941	0.2418	0.1851	0.085*	
H63B	0.1859	0.2517	0.2514	0.085*	
C64	0.15631 (11)	0.2475 (4)	0.1855 (3)	0.0407 (14)	
C65	0.13608 (12)	0.2912 (5)	0.2090 (3)	0.0519 (17)	
H65	0.1388	0.3368	0.2403	0.062*	
C66	0.11230 (12)	0.2686 (4)	0.1869 (3)	0.0474 (16)	
H66	0.0991	0.2971	0.2042	0.057*	
C67	0.10789 (11)	0.2031 (4)	0.1387 (3)	0.0396 (14)	
C68	0.12771 (11)	0.1586 (5)	0.1154 (3)	0.0448 (15)	
H68	0.1249	0.1138	0.0836	0.054*	
C69	0.15173 (11)	0.1799 (5)	0.1389 (3)	0.0458 (15)	
H69	0.1649	0.1486	0.1231	0.055*	
C70	0.06811 (12)	0.1462 (5)	0.1478 (3)	0.0505 (17)	
H70	0.0736	0.1344	0.1887	0.061*	
C71	0.04264 (12)	0.1231 (5)	0.1253 (3)	0.0501 (16)	
C72	0.02601 (13)	0.0834 (6)	0.1621 (4)	0.065 (2)	
H72	0.0309	0.0700	0.2031	0.078*	
C73	0.00194 (14)	0.0636 (6)	0.1376 (4)	0.073 (2)	
H73	−0.0095	0.0363	0.1616	0.088*	
C74	−0.00447 (13)	0.0850 (6)	0.0783 (4)	0.068 (2)	
H74	−0.0206	0.0734	0.0610	0.082*	
C75	0.01268 (12)	0.1237 (5)	0.0433 (3)	0.0542 (17)	
H75	0.0079	0.1373	0.0023	0.065*	
N1	0.17012 (9)	0.9418 (3)	0.3052 (2)	0.0393 (12)	
N2	0.15161 (8)	0.8470 (3)	0.2043 (2)	0.0369 (11)	
N3	0.09855 (8)	0.2609 (3)	−0.0198 (2)	0.0347 (11)	
N4	0.08002 (9)	0.0809 (3)	−0.0136 (2)	0.0425 (12)	
N5	0.21319 (9)	0.7815 (4)	0.3375 (2)	0.0457 (13)	
N6	0.16719 (9)	0.7052 (3)	0.3266 (2)	0.0406 (12)	
N7	0.04992 (9)	0.3525 (3)	0.0454 (2)	0.0359 (11)	
N8	0.04071 (9)	0.2498 (4)	−0.0584 (2)	0.0418 (12)	
N9	0.20538 (9)	0.8857 (4)	0.2062 (2)	0.0442 (12)	
N10	0.19739 (9)	0.6903 (4)	0.2056 (2)	0.0450 (13)	
N11	0.08297 (9)	0.1819 (3)	0.1130 (2)	0.0389 (11)	
N12	0.03601 (9)	0.1428 (4)	0.0656 (2)	0.0434 (12)	
B1	0.18599 (16)	0.8728 (7)	0.5005 (4)	0.060 (2)	
F1	0.17030 (9)	0.8358 (4)	0.4533 (3)	0.1042 (17)	
F2	0.20725 (8)	0.9030 (4)	0.4771 (2)	0.0831 (14)	
F3	0.17398 (12)	0.9460 (4)	0.5255 (3)	0.121 (2)	
F4	0.19219 (10)	0.8013 (5)	0.5429 (3)	0.126 (2)	
B2	0.0349 (2)	0.3612 (7)	0.4514 (6)	0.084 (3)	
F5	0.03959 (12)	0.4338 (4)	0.4130 (3)	0.134 (2)	
F6	0.03165 (16)	0.4014 (5)	0.5067 (4)	0.172 (3)	
F7	0.05406 (10)	0.2983 (4)	0.4564 (3)	0.1120 (19)	
F8	0.01419 (11)	0.3134 (5)	0.4329 (4)	0.159 (3)	
B3	0.0716 (3)	0.0533 (9)	0.7750 (6)	0.084 (3)	
F9A	0.0485 (5)	0.076 (4)	0.7657 (17)	0.25 (2)	0.5
F11A	0.0726 (9)	−0.0468 (11)	0.7839 (9)	0.194 (13)	0.5
F12A	0.0822 (5)	0.0620 (18)	0.7257 (8)	0.147 (9)	0.5
F9B	0.0583 (4)	0.1303 (12)	0.7483 (9)	0.120 (7)	0.5
F11B	0.0543 (5)	0.0037 (19)	0.7999 (11)	0.154 (9)	0.5
F12B	0.0843 (4)	0.009 (2)	0.7371 (19)	0.210 (19)	0.5
F10	0.08526 (11)	0.0978 (4)	0.8228 (3)	0.1140 (19)	
B4	0.2052 (3)	0.6248 (16)	0.9504 (10)	0.134 (6)	
F13	0.1957 (3)	0.5481 (8)	0.9320 (7)	0.287 (8)	
F14	0.19950 (15)	0.6357 (9)	1.0067 (4)	0.215 (5)	
F15	0.22659 (15)	0.6319 (10)	0.9394 (5)	0.247 (5)	
F16	0.1906 (2)	0.6894 (9)	0.9174 (7)	0.276 (7)	
N13	0.0960 (2)	0.2719 (7)	0.3333 (4)	0.110 (3)	
C76	0.0494 (2)	0.2407 (9)	0.3129 (6)	0.140 (5)	
H76A	0.0445	0.2474	0.2698	0.211*	
H76B	0.0408	0.2881	0.3351	0.211*	
H76C	0.0454	0.1762	0.3260	0.211*	
C77	0.0760 (3)	0.2564 (8)	0.3247 (4)	0.095 (3)	
N14	0.2643 (3)	0.6288 (19)	0.1853 (9)	0.239 (10)	
C78	0.2615 (3)	0.6122 (13)	0.0704 (8)	0.194 (8)	
H78A	0.2631	0.6757	0.0527	0.291*	
H78B	0.2458	0.5846	0.0556	0.291*	
H78C	0.2746	0.5706	0.0589	0.291*	
C79	0.2632 (3)	0.6203 (17)	0.1355 (11)	0.173 (9)	
N15	0.03167 (16)	0.8665 (6)	0.0752 (4)	0.104 (3)	
C80	0.0639 (2)	0.7454 (10)	0.1190 (6)	0.155 (5)	
H80A	0.0794	0.7601	0.1033	0.233*	
H80B	0.0588	0.6805	0.1069	0.233*	
H80C	0.0659	0.7496	0.1629	0.233*	
C81	0.04554 (19)	0.8137 (7)	0.0951 (4)	0.086 (3)	

### Atomic displacement parameters (Å^2^)

**Table:**

	*U*^11^	*U*^22^	*U*^33^	*U*^12^	*U*^13^	*U*^23^
Zn1	0.0400 (4)	0.0352 (4)	0.0402 (4)	−0.0020 (3)	−0.0009 (3)	0.0029 (3)
Zn2	0.0382 (4)	0.0339 (4)	0.0417 (4)	0.0022 (3)	0.0000 (3)	−0.0027 (3)
C1	0.048 (4)	0.033 (3)	0.035 (3)	−0.002 (3)	0.004 (3)	0.001 (3)
C2	0.058 (4)	0.044 (4)	0.050 (4)	0.002 (3)	0.004 (3)	0.000 (3)
C3	0.085 (6)	0.041 (4)	0.056 (5)	0.002 (4)	0.022 (4)	−0.007 (3)
C4	0.073 (5)	0.064 (5)	0.049 (4)	−0.010 (4)	0.014 (4)	−0.022 (4)
C5	0.051 (4)	0.054 (4)	0.043 (4)	0.003 (3)	0.004 (3)	−0.001 (3)
C6	0.048 (4)	0.033 (3)	0.045 (4)	0.001 (3)	−0.001 (3)	0.007 (3)
C7	0.037 (3)	0.038 (3)	0.040 (3)	−0.001 (3)	−0.003 (3)	0.001 (3)
C8	0.070 (4)	0.042 (4)	0.037 (3)	−0.019 (3)	0.011 (3)	0.003 (3)
C9	0.063 (4)	0.031 (3)	0.048 (4)	−0.016 (3)	0.004 (3)	−0.004 (3)
C10	0.043 (3)	0.036 (3)	0.036 (3)	0.000 (3)	−0.003 (3)	0.006 (3)
C11	0.076 (5)	0.041 (4)	0.041 (4)	−0.017 (3)	0.003 (3)	0.005 (3)
C12	0.071 (5)	0.030 (3)	0.051 (4)	−0.016 (3)	−0.003 (3)	0.004 (3)
C13	0.070 (5)	0.042 (4)	0.040 (4)	−0.007 (3)	0.004 (3)	−0.002 (3)
C14	0.052 (4)	0.032 (3)	0.027 (3)	−0.002 (3)	0.003 (3)	−0.002 (2)
C15	0.038 (3)	0.050 (4)	0.044 (4)	−0.007 (3)	0.001 (3)	−0.002 (3)
C16	0.043 (4)	0.038 (3)	0.047 (4)	0.004 (3)	−0.009 (3)	0.003 (3)
C17	0.042 (3)	0.030 (3)	0.030 (3)	−0.002 (3)	0.010 (3)	−0.003 (2)
C18	0.032 (3)	0.039 (3)	0.038 (3)	0.001 (3)	0.004 (3)	0.002 (3)
C19	0.059 (4)	0.033 (3)	0.034 (3)	0.009 (3)	0.008 (3)	0.003 (3)
C20	0.045 (3)	0.033 (3)	0.034 (3)	0.000 (3)	0.000 (3)	0.001 (3)
C21	0.039 (3)	0.030 (3)	0.039 (3)	0.006 (3)	−0.005 (3)	−0.003 (3)
C22	0.054 (4)	0.044 (4)	0.065 (5)	0.007 (3)	0.012 (3)	−0.007 (3)
C23	0.066 (5)	0.037 (4)	0.093 (6)	0.009 (4)	0.004 (4)	−0.015 (4)
C24	0.073 (5)	0.031 (4)	0.093 (6)	−0.004 (4)	0.002 (5)	−0.015 (4)
C25	0.045 (4)	0.043 (4)	0.073 (5)	−0.007 (3)	0.001 (3)	−0.008 (3)
C26	0.060 (4)	0.044 (4)	0.034 (3)	0.012 (3)	−0.003 (3)	−0.011 (3)
C27	0.067 (5)	0.065 (5)	0.044 (4)	0.026 (4)	−0.011 (4)	−0.007 (3)
C28	0.060 (5)	0.080 (6)	0.056 (5)	0.031 (4)	−0.015 (4)	−0.017 (4)
C29	0.037 (4)	0.090 (6)	0.085 (6)	0.009 (4)	−0.008 (4)	−0.026 (5)
C30	0.045 (4)	0.071 (5)	0.065 (5)	−0.002 (4)	−0.007 (4)	−0.009 (4)
C31	0.054 (4)	0.042 (4)	0.034 (3)	0.004 (3)	0.001 (3)	−0.001 (3)
C32	0.044 (3)	0.036 (3)	0.030 (3)	−0.001 (3)	−0.001 (3)	−0.001 (3)
C33	0.052 (4)	0.036 (3)	0.043 (4)	0.001 (3)	0.011 (3)	0.002 (3)
C34	0.063 (5)	0.035 (4)	0.057 (4)	−0.010 (3)	0.016 (3)	−0.003 (3)
C35	0.046 (4)	0.050 (4)	0.032 (3)	−0.007 (3)	0.009 (3)	−0.010 (3)
C36	0.050 (4)	0.047 (4)	0.040 (4)	0.006 (3)	0.003 (3)	0.000 (3)
C37	0.050 (4)	0.034 (3)	0.035 (3)	−0.001 (3)	0.006 (3)	0.005 (3)
C38	0.050 (4)	0.065 (5)	0.048 (4)	−0.011 (3)	0.017 (3)	−0.012 (3)
C39	0.043 (4)	0.037 (3)	0.041 (3)	0.003 (3)	0.006 (3)	−0.005 (3)
C40	0.051 (4)	0.078 (5)	0.054 (4)	−0.010 (4)	0.023 (3)	−0.021 (4)
C41	0.047 (4)	0.066 (5)	0.057 (4)	−0.015 (3)	0.009 (3)	−0.015 (4)
C42	0.045 (4)	0.031 (3)	0.041 (3)	0.002 (3)	0.003 (3)	0.001 (3)
C43	0.048 (4)	0.056 (4)	0.045 (4)	−0.006 (3)	0.014 (3)	−0.007 (3)
C44	0.051 (4)	0.043 (4)	0.047 (4)	−0.016 (3)	0.008 (3)	−0.010 (3)
C45	0.051 (4)	0.032 (3)	0.051 (4)	0.002 (3)	0.014 (3)	−0.002 (3)
C46	0.035 (3)	0.044 (4)	0.044 (4)	0.001 (3)	0.003 (3)	−0.001 (3)
C47	0.053 (4)	0.062 (5)	0.059 (5)	0.018 (4)	−0.002 (3)	0.005 (4)
C48	0.077 (5)	0.093 (6)	0.043 (4)	0.010 (5)	−0.013 (4)	0.007 (4)
C49	0.081 (6)	0.084 (6)	0.043 (4)	0.012 (5)	−0.008 (4)	−0.013 (4)
C50	0.058 (4)	0.067 (5)	0.054 (4)	0.007 (4)	0.002 (4)	−0.014 (4)
C51	0.049 (4)	0.046 (4)	0.065 (5)	0.008 (3)	0.022 (3)	0.006 (3)
C52	0.110 (7)	0.050 (5)	0.088 (6)	−0.003 (5)	0.056 (5)	0.004 (4)
C53	0.104 (7)	0.060 (5)	0.112 (7)	−0.003 (5)	0.059 (6)	0.029 (5)
C54	0.094 (6)	0.035 (4)	0.116 (7)	−0.007 (4)	0.047 (6)	0.008 (4)
C55	0.063 (5)	0.041 (4)	0.077 (5)	−0.004 (3)	0.011 (4)	0.003 (4)
C56	0.055 (4)	0.040 (4)	0.062 (5)	0.002 (3)	0.021 (4)	−0.004 (3)
C57	0.044 (4)	0.038 (3)	0.048 (4)	0.002 (3)	0.009 (3)	0.004 (3)
C58	0.070 (5)	0.044 (4)	0.053 (4)	−0.005 (3)	−0.010 (4)	0.007 (3)
C59	0.074 (5)	0.047 (4)	0.060 (5)	−0.011 (4)	−0.023 (4)	0.000 (4)
C60	0.034 (3)	0.038 (4)	0.062 (4)	−0.004 (3)	−0.003 (3)	0.005 (3)
C61	0.052 (4)	0.038 (4)	0.051 (4)	0.010 (3)	0.003 (3)	0.005 (3)
C62	0.053 (4)	0.046 (4)	0.050 (4)	0.002 (3)	−0.001 (3)	−0.009 (3)
C63	0.044 (4)	0.037 (4)	0.087 (5)	0.000 (3)	−0.004 (4)	0.004 (3)
C64	0.038 (3)	0.029 (3)	0.055 (4)	−0.002 (3)	0.004 (3)	0.007 (3)
C65	0.051 (4)	0.039 (4)	0.064 (4)	0.001 (3)	−0.005 (3)	−0.012 (3)
C66	0.044 (4)	0.043 (4)	0.056 (4)	0.008 (3)	0.003 (3)	0.000 (3)
C67	0.040 (3)	0.040 (3)	0.039 (3)	−0.005 (3)	0.001 (3)	0.009 (3)
C68	0.048 (4)	0.046 (4)	0.040 (4)	0.001 (3)	0.003 (3)	0.002 (3)
C69	0.041 (4)	0.048 (4)	0.048 (4)	0.006 (3)	0.006 (3)	0.009 (3)
C70	0.045 (4)	0.055 (4)	0.050 (4)	0.004 (3)	−0.007 (3)	0.010 (3)
C71	0.051 (4)	0.047 (4)	0.053 (4)	−0.005 (3)	0.007 (3)	0.005 (3)
C72	0.058 (5)	0.075 (5)	0.064 (5)	−0.003 (4)	0.009 (4)	0.013 (4)
C73	0.044 (4)	0.078 (6)	0.101 (7)	−0.017 (4)	0.017 (4)	0.005 (5)
C74	0.038 (4)	0.072 (5)	0.093 (6)	0.001 (4)	−0.007 (4)	−0.002 (5)
C75	0.046 (4)	0.051 (4)	0.064 (5)	0.000 (3)	−0.001 (3)	−0.001 (3)
N1	0.045 (3)	0.040 (3)	0.031 (3)	−0.008 (2)	−0.001 (2)	−0.003 (2)
N2	0.038 (3)	0.037 (3)	0.034 (3)	−0.006 (2)	−0.002 (2)	0.003 (2)
N3	0.043 (3)	0.030 (3)	0.031 (3)	0.002 (2)	0.002 (2)	−0.001 (2)
N4	0.045 (3)	0.032 (3)	0.050 (3)	−0.004 (2)	−0.001 (2)	−0.004 (2)
N5	0.039 (3)	0.047 (3)	0.049 (3)	0.002 (2)	−0.006 (2)	−0.005 (3)
N6	0.050 (3)	0.038 (3)	0.034 (3)	0.004 (2)	0.002 (2)	−0.006 (2)
N7	0.037 (3)	0.036 (3)	0.033 (3)	0.003 (2)	−0.002 (2)	−0.001 (2)
N8	0.043 (3)	0.044 (3)	0.038 (3)	0.000 (2)	0.005 (2)	−0.006 (2)
N9	0.042 (3)	0.035 (3)	0.056 (3)	−0.002 (2)	0.007 (3)	0.006 (3)
N10	0.037 (3)	0.040 (3)	0.057 (3)	0.004 (2)	0.003 (3)	0.007 (3)
N11	0.038 (3)	0.036 (3)	0.041 (3)	0.001 (2)	0.001 (2)	0.003 (2)
N12	0.031 (3)	0.041 (3)	0.056 (3)	0.000 (2)	−0.002 (2)	0.003 (3)
B1	0.051 (5)	0.072 (6)	0.058 (5)	0.004 (5)	0.007 (4)	0.005 (5)
F1	0.070 (3)	0.125 (4)	0.117 (4)	−0.025 (3)	0.007 (3)	−0.032 (4)
F2	0.061 (3)	0.112 (4)	0.074 (3)	−0.016 (3)	−0.002 (2)	0.017 (3)
F3	0.146 (5)	0.108 (4)	0.112 (4)	0.048 (4)	0.031 (4)	−0.018 (4)
F4	0.094 (4)	0.160 (6)	0.131 (5)	0.050 (4)	0.046 (3)	0.081 (4)
B2	0.067 (7)	0.049 (6)	0.138 (11)	0.012 (5)	0.025 (7)	−0.008 (7)
F5	0.135 (5)	0.084 (4)	0.186 (6)	0.018 (4)	0.033 (5)	0.051 (4)
F6	0.239 (9)	0.098 (5)	0.192 (7)	0.019 (5)	0.092 (7)	−0.026 (5)
F7	0.093 (4)	0.085 (4)	0.161 (5)	0.035 (3)	0.029 (4)	0.014 (4)
F8	0.071 (4)	0.134 (6)	0.267 (9)	−0.019 (4)	−0.007 (5)	−0.006 (6)
B3	0.086 (9)	0.075 (8)	0.088 (9)	−0.015 (7)	−0.001 (7)	−0.018 (7)
F9A	0.093 (15)	0.37 (5)	0.27 (4)	0.05 (2)	−0.020 (18)	−0.25 (4)
F11A	0.38 (4)	0.061 (10)	0.130 (14)	−0.055 (16)	−0.049 (19)	−0.012 (9)
F12A	0.19 (2)	0.17 (2)	0.094 (11)	0.002 (17)	0.064 (11)	−0.002 (12)
F9B	0.112 (14)	0.111 (11)	0.124 (12)	0.004 (9)	−0.048 (10)	0.024 (10)
F11B	0.132 (18)	0.116 (17)	0.21 (2)	−0.062 (13)	0.030 (14)	0.006 (15)
F12B	0.086 (10)	0.17 (2)	0.38 (4)	0.013 (14)	0.037 (18)	−0.20 (3)
F10	0.141 (5)	0.087 (4)	0.104 (4)	−0.025 (3)	−0.038 (4)	−0.003 (3)
B4	0.088 (10)	0.152 (16)	0.170 (17)	0.019 (10)	0.054 (11)	0.067 (14)
F13	0.329 (16)	0.151 (9)	0.406 (19)	−0.092 (10)	0.163 (14)	−0.109 (11)
F14	0.137 (7)	0.395 (16)	0.115 (6)	0.023 (8)	0.020 (5)	−0.038 (8)
F15	0.094 (6)	0.398 (16)	0.260 (11)	−0.012 (8)	0.079 (6)	−0.008 (11)
F16	0.239 (12)	0.258 (13)	0.347 (16)	0.073 (10)	0.113 (11)	0.146 (12)
N13	0.134 (8)	0.099 (7)	0.097 (7)	−0.013 (7)	0.017 (7)	−0.001 (5)
C76	0.153 (12)	0.118 (10)	0.145 (11)	−0.024 (9)	−0.009 (9)	−0.023 (8)
C77	0.154 (11)	0.076 (7)	0.056 (6)	−0.024 (8)	0.016 (7)	−0.008 (5)
N14	0.164 (13)	0.34 (2)	0.208 (17)	0.115 (14)	0.017 (14)	0.11 (2)
C78	0.160 (14)	0.204 (17)	0.203 (18)	0.091 (12)	−0.057 (14)	−0.015 (15)
C79	0.092 (10)	0.229 (19)	0.20 (2)	0.083 (11)	0.008 (14)	0.09 (2)
N15	0.105 (7)	0.092 (6)	0.109 (7)	0.013 (5)	−0.011 (5)	0.000 (5)
C80	0.150 (11)	0.159 (12)	0.146 (11)	0.074 (10)	−0.035 (9)	0.021 (9)
C81	0.087 (7)	0.084 (7)	0.085 (7)	0.006 (6)	−0.005 (5)	0.004 (5)

### Geometric parameters (Å, º)

**Table:**

Zn1—N5	2.128 (5)	C43—H43	0.9300
Zn1—N6	2.157 (5)	C44—H44	0.9300
Zn1—N9	2.160 (5)	C45—N7	1.261 (7)
Zn1—N1	2.172 (5)	C45—C46	1.475 (8)
Zn1—N2	2.185 (5)	C45—H45	0.9300
Zn1—N10	2.277 (5)	C46—N8	1.335 (7)
Zn2—N4	2.135 (5)	C46—C47	1.376 (8)
Zn2—N8	2.145 (5)	C47—C48	1.377 (10)
Zn2—N3	2.169 (5)	C47—H47	0.9300
Zn2—N7	2.183 (5)	C48—C49	1.370 (10)
Zn2—N12	2.190 (5)	C48—H48	0.9300
Zn2—N11	2.192 (5)	C49—C50	1.370 (10)
C1—N1	1.353 (7)	C49—H49	0.9300
C1—C2	1.366 (8)	C50—N8	1.346 (8)
C1—C6	1.455 (8)	C50—H50	0.9300
C2—C3	1.383 (9)	C51—N9	1.344 (8)
C2—H2	0.9300	C51—C52	1.370 (9)
C3—C4	1.371 (10)	C51—C56	1.463 (9)
C3—H3	0.9300	C52—C53	1.393 (11)
C4—C5	1.401 (9)	C52—H52	0.9300
C4—H4	0.9300	C53—C54	1.364 (11)
C5—N1	1.328 (7)	C53—H53	0.9300
C5—H5	0.9300	C54—C55	1.371 (10)
C6—N2	1.267 (7)	C54—H54	0.9300
C6—H6	0.9300	C55—N9	1.334 (8)
C7—C12	1.367 (8)	C55—H55	0.9300
C7—C8	1.379 (8)	C56—N10	1.248 (8)
C7—N2	1.438 (7)	C56—H56	0.9300
C8—C9	1.387 (8)	C57—C62	1.373 (8)
C8—H8	0.9300	C57—C58	1.386 (9)
C9—C10	1.372 (8)	C57—N10	1.445 (7)
C9—H9	0.9300	C58—C59	1.375 (9)
C10—C11	1.382 (8)	C58—H58	0.9300
C10—C13	1.520 (8)	C59—C60	1.377 (9)
C11—C12	1.374 (9)	C59—H59	0.9300
C11—H11	0.9300	C60—C61	1.366 (9)
C12—H12	0.9300	C60—C63	1.521 (8)
C13—C14	1.512 (8)	C61—C62	1.379 (9)
C13—H13A	0.9700	C61—H61	0.9300
C13—H13B	0.9700	C62—H62	0.9300
C14—C15	1.376 (8)	C63—C64	1.515 (8)
C14—C19	1.379 (8)	C63—H63A	0.9700
C15—C16	1.387 (8)	C63—H63B	0.9700
C15—H15	0.9300	C64—C69	1.383 (8)
C16—C17	1.368 (8)	C64—C65	1.392 (8)
C16—H16	0.9300	C65—C66	1.366 (8)
C17—C18	1.384 (8)	C65—H65	0.9300
C17—N3	1.447 (7)	C66—C67	1.389 (8)
C18—C19	1.388 (8)	C66—H66	0.9300
C18—H18	0.9300	C67—C68	1.376 (8)
C19—H19	0.9300	C67—N11	1.440 (7)
C20—N3	1.278 (7)	C68—C69	1.382 (8)
C20—C21	1.463 (8)	C68—H68	0.9300
C20—H20	0.9300	C69—H69	0.9300
C21—N4	1.345 (7)	C70—N11	1.261 (8)
C21—C22	1.376 (8)	C70—C71	1.453 (9)
C22—C23	1.381 (9)	C70—H70	0.9300
C22—H22	0.9300	C71—N12	1.350 (8)
C23—C24	1.346 (10)	C71—C72	1.379 (9)
C23—H23	0.9300	C72—C73	1.387 (10)
C24—C25	1.386 (9)	C72—H72	0.9300
C24—H24	0.9300	C73—C74	1.345 (11)
C25—N4	1.327 (8)	C73—H73	0.9300
C25—H25	0.9300	C74—C75	1.369 (10)
C26—N5	1.338 (8)	C74—H74	0.9300
C26—C27	1.371 (9)	C75—N12	1.335 (8)
C26—C31	1.464 (9)	C75—H75	0.9300
C27—C28	1.376 (10)	B1—F3	1.342 (10)
C27—H27	0.9300	B1—F4	1.369 (10)
C28—C29	1.349 (11)	B1—F1	1.369 (10)
C28—H28	0.9300	B1—F2	1.370 (9)
C29—C30	1.379 (10)	B2—F8	1.326 (12)
C29—H29	0.9300	B2—F5	1.343 (12)
C30—N5	1.332 (8)	B2—F7	1.346 (10)
C30—H30	0.9300	B2—F6	1.360 (13)
C31—N6	1.280 (7)	B3—F12A	1.28 (2)
C31—H31	0.9300	B3—F9A	1.28 (2)
C32—C37	1.388 (8)	B3—F12B	1.28 (3)
C32—C33	1.390 (8)	B3—F11B	1.32 (2)
C32—N6	1.429 (7)	B3—F10	1.366 (11)
C33—C34	1.356 (8)	B3—F9B	1.37 (2)
C33—H33	0.9300	B3—F11A	1.39 (2)
C34—C35	1.396 (9)	B4—F15	1.213 (15)
C34—H34	0.9300	B4—F13	1.22 (2)
C35—C36	1.376 (8)	B4—F14	1.310 (19)
C35—C38	1.509 (8)	B4—F16	1.348 (17)
C36—C37	1.376 (8)	N13—C77	1.104 (13)
C36—H36	0.9300	C76—C77	1.453 (16)
C37—H37	0.9300	C76—H76A	0.9600
C38—C39	1.520 (8)	C76—H76B	0.9600
C38—H38A	0.9700	C76—H76C	0.9600
C38—H38B	0.9700	N14—C79	1.09 (2)
C39—C40	1.380 (8)	C78—C79	1.43 (2)
C39—C44	1.382 (8)	C78—H78A	0.9600
C40—C41	1.385 (9)	C78—H78B	0.9600
C40—H40	0.9300	C78—H78C	0.9600
C41—C42	1.364 (8)	N15—C81	1.102 (10)
C41—H41	0.9300	C80—C81	1.426 (13)
C42—C43	1.373 (8)	C80—H80A	0.9600
C42—N7	1.445 (7)	C80—H80B	0.9600
C43—C44	1.385 (8)	C80—H80C	0.9600
			
N5—Zn1—N6	77.8 (2)	C48—C47—H47	120.5
N5—Zn1—N9	96.7 (2)	C49—C48—C47	118.6 (7)
N6—Zn1—N9	168.32 (19)	C49—C48—H48	120.7
N5—Zn1—N1	97.40 (18)	C47—C48—H48	120.7
N6—Zn1—N1	98.47 (18)	C50—C49—C48	119.7 (7)
N9—Zn1—N1	92.43 (18)	C50—C49—H49	120.2
N5—Zn1—N2	172.77 (19)	C48—C49—H49	120.2
N6—Zn1—N2	100.96 (18)	N8—C50—C49	122.1 (7)
N9—Zn1—N2	85.75 (18)	N8—C50—H50	118.9
N1—Zn1—N2	75.67 (18)	C49—C50—H50	118.9
N5—Zn1—N10	92.72 (18)	N9—C51—C52	122.8 (6)
N6—Zn1—N10	94.60 (18)	N9—C51—C56	115.6 (6)
N9—Zn1—N10	75.21 (19)	C52—C51—C56	121.6 (7)
N1—Zn1—N10	164.89 (18)	C51—C52—C53	118.7 (8)
N2—Zn1—N10	94.48 (18)	C51—C52—H52	120.7
N4—Zn2—N8	97.97 (19)	C53—C52—H52	120.7
N4—Zn2—N3	76.67 (18)	C54—C53—C52	118.4 (7)
N8—Zn2—N3	94.45 (18)	C54—C53—H53	120.8
N4—Zn2—N7	174.00 (18)	C52—C53—H53	120.8
N8—Zn2—N7	76.51 (18)	C53—C54—C55	119.7 (7)
N3—Zn2—N7	101.20 (17)	C53—C54—H54	120.2
N4—Zn2—N12	95.52 (19)	C55—C54—H54	120.2
N8—Zn2—N12	92.12 (18)	N9—C55—C54	122.7 (7)
N3—Zn2—N12	170.41 (17)	N9—C55—H55	118.7
N7—Zn2—N12	87.12 (18)	C54—C55—H55	118.7
N4—Zn2—N11	91.25 (18)	N10—C56—C51	121.9 (6)
N8—Zn2—N11	165.82 (18)	N10—C56—H56	119.1
N3—Zn2—N11	98.15 (17)	C51—C56—H56	119.1
N7—Zn2—N11	94.61 (17)	C62—C57—C58	118.6 (6)
N12—Zn2—N11	76.22 (18)	C62—C57—N10	119.5 (6)
N1—C1—C2	122.3 (6)	C58—C57—N10	121.9 (6)
N1—C1—C6	114.9 (5)	C59—C58—C57	119.8 (6)
C2—C1—C6	122.7 (6)	C59—C58—H58	120.1
C1—C2—C3	120.3 (6)	C57—C58—H58	120.1
C1—C2—H2	119.9	C58—C59—C60	121.5 (6)
C3—C2—H2	119.9	C58—C59—H59	119.2
C4—C3—C2	117.9 (7)	C60—C59—H59	119.2
C4—C3—H3	121.1	C61—C60—C59	118.2 (6)
C2—C3—H3	121.1	C61—C60—C63	121.5 (6)
C3—C4—C5	119.1 (6)	C59—C60—C63	120.3 (6)
C3—C4—H4	120.4	C60—C61—C62	121.0 (6)
C5—C4—H4	120.4	C60—C61—H61	119.5
N1—C5—C4	122.7 (6)	C62—C61—H61	119.5
N1—C5—H5	118.6	C57—C62—C61	120.8 (6)
C4—C5—H5	118.6	C57—C62—H62	119.6
N2—C6—C1	120.5 (6)	C61—C62—H62	119.6
N2—C6—H6	119.7	C64—C63—C60	112.8 (5)
C1—C6—H6	119.7	C64—C63—H63A	109.0
C12—C7—C8	119.9 (6)	C60—C63—H63A	109.0
C12—C7—N2	119.4 (5)	C64—C63—H63B	109.0
C8—C7—N2	120.4 (5)	C60—C63—H63B	109.0
C7—C8—C9	119.2 (6)	H63A—C63—H63B	107.8
C7—C8—H8	120.4	C69—C64—C65	118.1 (6)
C9—C8—H8	120.4	C69—C64—C63	121.0 (6)
C10—C9—C8	121.8 (6)	C65—C64—C63	120.9 (6)
C10—C9—H9	119.1	C66—C65—C64	121.4 (6)
C8—C9—H9	119.1	C66—C65—H65	119.3
C9—C10—C11	117.3 (6)	C64—C65—H65	119.3
C9—C10—C13	123.4 (5)	C65—C66—C67	120.0 (6)
C11—C10—C13	119.3 (5)	C65—C66—H66	120.0
C12—C11—C10	121.9 (6)	C67—C66—H66	120.0
C12—C11—H11	119.1	C68—C67—C66	119.2 (6)
C10—C11—H11	119.1	C68—C67—N11	120.1 (5)
C7—C12—C11	119.8 (6)	C66—C67—N11	120.8 (5)
C7—C12—H12	120.1	C67—C68—C69	120.5 (6)
C11—C12—H12	120.1	C67—C68—H68	119.7
C14—C13—C10	114.9 (5)	C69—C68—H68	119.7
C14—C13—H13A	108.6	C68—C69—C64	120.7 (6)
C10—C13—H13A	108.6	C68—C69—H69	119.7
C14—C13—H13B	108.6	C64—C69—H69	119.7
C10—C13—H13B	108.6	N11—C70—C71	121.4 (6)
H13A—C13—H13B	107.5	N11—C70—H70	119.3
C15—C14—C19	118.9 (5)	C71—C70—H70	119.3
C15—C14—C13	121.0 (6)	N12—C71—C72	121.3 (6)
C19—C14—C13	120.1 (5)	N12—C71—C70	115.9 (6)
C14—C15—C16	121.5 (6)	C72—C71—C70	122.8 (7)
C14—C15—H15	119.2	C71—C72—C73	119.7 (7)
C16—C15—H15	119.2	C71—C72—H72	120.2
C17—C16—C15	118.9 (6)	C73—C72—H72	120.2
C17—C16—H16	120.5	C74—C73—C72	118.3 (7)
C15—C16—H16	120.5	C74—C73—H73	120.8
C16—C17—C18	120.7 (5)	C72—C73—H73	120.8
C16—C17—N3	122.4 (5)	C73—C74—C75	120.1 (7)
C18—C17—N3	116.9 (5)	C73—C74—H74	119.9
C17—C18—C19	119.6 (5)	C75—C74—H74	119.9
C17—C18—H18	120.2	N12—C75—C74	122.8 (7)
C19—C18—H18	120.2	N12—C75—H75	118.6
C14—C19—C18	120.3 (5)	C74—C75—H75	118.6
C14—C19—H19	119.9	C5—N1—C1	117.6 (5)
C18—C19—H19	119.9	C5—N1—Zn1	127.7 (4)
N3—C20—C21	119.3 (5)	C1—N1—Zn1	114.5 (4)
N3—C20—H20	120.3	C6—N2—C7	119.7 (5)
C21—C20—H20	120.3	C6—N2—Zn1	114.1 (4)
N4—C21—C22	123.1 (6)	C7—N2—Zn1	124.3 (4)
N4—C21—C20	115.5 (5)	C20—N3—C17	117.7 (5)
C22—C21—C20	121.4 (6)	C20—N3—Zn2	113.9 (4)
C21—C22—C23	117.9 (6)	C17—N3—Zn2	128.3 (3)
C21—C22—H22	121.0	C25—N4—C21	117.3 (5)
C23—C22—H22	121.0	C25—N4—Zn2	128.2 (5)
C24—C23—C22	120.0 (7)	C21—N4—Zn2	114.5 (4)
C24—C23—H23	120.0	C30—N5—C26	117.4 (6)
C22—C23—H23	120.0	C30—N5—Zn1	128.6 (5)
C23—C24—C25	118.8 (7)	C26—N5—Zn1	113.7 (4)
C23—C24—H24	120.6	C31—N6—C32	118.9 (5)
C25—C24—H24	120.6	C31—N6—Zn1	112.3 (4)
N4—C25—C24	122.9 (7)	C32—N6—Zn1	128.3 (4)
N4—C25—H25	118.5	C45—N7—C42	118.4 (5)
C24—C25—H25	118.5	C45—N7—Zn2	112.6 (4)
N5—C26—C27	123.2 (7)	C42—N7—Zn2	127.4 (4)
N5—C26—C31	115.6 (5)	C46—N8—C50	117.8 (6)
C27—C26—C31	121.2 (7)	C46—N8—Zn2	114.2 (4)
C26—C27—C28	118.3 (7)	C50—N8—Zn2	127.9 (5)
C26—C27—H27	120.9	C55—N9—C51	117.7 (6)
C28—C27—H27	120.9	C55—N9—Zn1	127.3 (5)
C29—C28—C27	119.2 (7)	C51—N9—Zn1	114.8 (4)
C29—C28—H28	120.4	C56—N10—C57	117.7 (6)
C27—C28—H28	120.4	C56—N10—Zn1	110.9 (4)
C28—C29—C30	119.7 (7)	C57—N10—Zn1	130.8 (4)
C28—C29—H29	120.2	C70—N11—C67	118.2 (5)
C30—C29—H29	120.2	C70—N11—Zn2	113.2 (4)
N5—C30—C29	122.2 (8)	C67—N11—Zn2	128.5 (4)
N5—C30—H30	118.9	C75—N12—C71	117.8 (6)
C29—C30—H30	118.9	C75—N12—Zn2	129.1 (5)
N6—C31—C26	120.4 (6)	C71—N12—Zn2	113.0 (4)
N6—C31—H31	119.8	F3—B1—F4	110.6 (7)
C26—C31—H31	119.8	F3—B1—F1	107.2 (7)
C37—C32—C33	119.1 (6)	F4—B1—F1	110.0 (8)
C37—C32—N6	118.6 (5)	F3—B1—F2	112.8 (8)
C33—C32—N6	122.3 (5)	F4—B1—F2	108.2 (7)
C34—C33—C32	119.5 (6)	F1—B1—F2	108.1 (7)
C34—C33—H33	120.3	F8—B2—F5	112.4 (11)
C32—C33—H33	120.3	F8—B2—F7	109.4 (8)
C33—C34—C35	122.7 (6)	F5—B2—F7	109.4 (9)
C33—C34—H34	118.6	F8—B2—F6	107.0 (10)
C35—C34—H34	118.6	F5—B2—F6	108.0 (8)
C36—C35—C34	116.9 (6)	F7—B2—F6	110.6 (11)
C36—C35—C38	121.5 (6)	F12A—B3—F9A	111 (2)
C34—C35—C38	121.7 (6)	F12B—B3—F11B	118.4 (18)
C35—C36—C37	121.7 (6)	F12A—B3—F10	110.4 (15)
C35—C36—H36	119.1	F9A—B3—F10	117.4 (14)
C37—C36—H36	119.1	F12B—B3—F10	114.8 (15)
C36—C37—C32	120.1 (6)	F11B—B3—F10	105.4 (15)
C36—C37—H37	120.0	F12B—B3—F9B	112 (2)
C32—C37—H37	120.0	F11B—B3—F9B	102.1 (18)
C35—C38—C39	115.6 (5)	F10—B3—F9B	101.8 (11)
C35—C38—H38A	108.4	F12A—B3—F11A	101.3 (19)
C39—C38—H38A	108.4	F9A—B3—F11A	107 (2)
C35—C38—H38B	108.4	F10—B3—F11A	108.8 (13)
C39—C38—H38B	108.4	F15—B4—F13	113 (2)
H38A—C38—H38B	107.4	F15—B4—F14	120 (2)
C40—C39—C44	116.7 (6)	F13—B4—F14	106.2 (14)
C40—C39—C38	120.0 (5)	F15—B4—F16	111.2 (15)
C44—C39—C38	123.3 (5)	F13—B4—F16	100.5 (18)
C39—C40—C41	122.8 (6)	F14—B4—F16	104.5 (16)
C39—C40—H40	118.6	C77—C76—H76A	109.5
C41—C40—H40	118.6	C77—C76—H76B	109.5
C42—C41—C40	118.9 (6)	H76A—C76—H76B	109.5
C42—C41—H41	120.5	C77—C76—H76C	109.5
C40—C41—H41	120.5	H76A—C76—H76C	109.5
C41—C42—C43	120.1 (6)	H76B—C76—H76C	109.5
C41—C42—N7	121.2 (5)	N13—C77—C76	177.4 (14)
C43—C42—N7	118.7 (5)	C79—C78—H78A	109.5
C42—C43—C44	120.1 (6)	C79—C78—H78B	109.5
C42—C43—H43	119.9	H78A—C78—H78B	109.5
C44—C43—H43	119.9	C79—C78—H78C	109.5
C39—C44—C43	121.3 (6)	H78A—C78—H78C	109.5
C39—C44—H44	119.3	H78B—C78—H78C	109.5
C43—C44—H44	119.3	N14—C79—C78	178 (3)
N7—C45—C46	120.0 (6)	C81—C80—H80A	109.5
N7—C45—H45	120.0	C81—C80—H80B	109.5
C46—C45—H45	120.0	H80A—C80—H80B	109.5
N8—C46—C47	122.8 (6)	C81—C80—H80C	109.5
N8—C46—C45	115.1 (5)	H80A—C80—H80C	109.5
C47—C46—C45	122.1 (6)	H80B—C80—H80C	109.5
C46—C47—C48	118.9 (7)	N15—C81—C80	178.0 (13)
C46—C47—H47	120.5		
