# Supplementary figures and images for: Crystal structure of tris­(μ-bis­{4-[(pyridin-2-yl­methyl­idene)amino]­phen­yl}methane-κ4 N,N′:N′′,N′′′)dizinc tetra­kis­(tetra­fluorido­borate) aceto­nitrile tris­olvate
Source: Acta Crystallogr E Crystallogr Commun. 2015 Dec 31;71(Pt 12):m271–2. doi: 10.1107/S205698901502455X (PMC4719869; doi:10.1107/S205698901502455X)

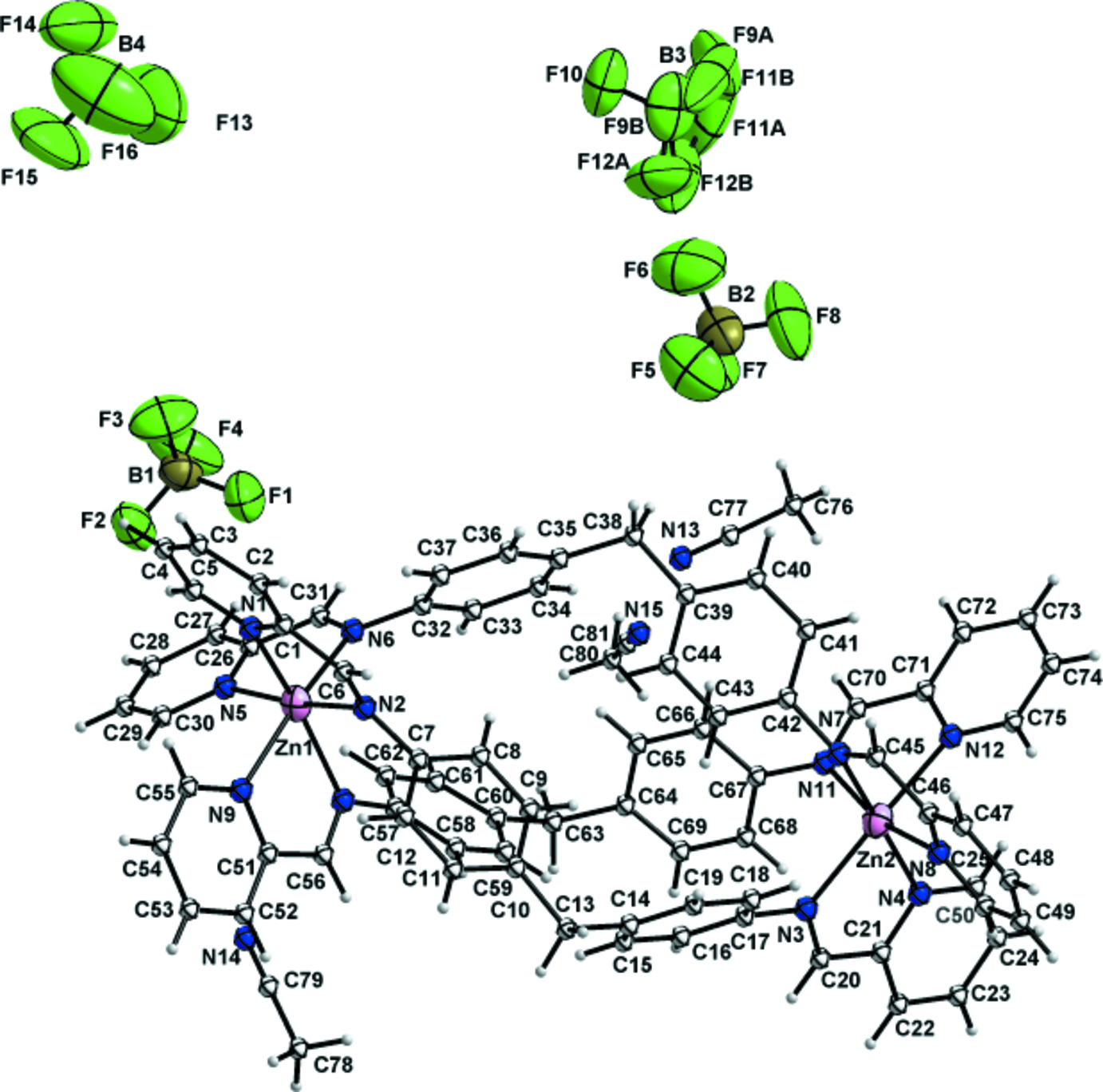

Supplement: Supplementary file 3 [file e-71-0m271-fig1.tif]
